# Assessing and Achieving Quality in Qualitative Research: Clues for Researchers in Training^*^

**DOI:** 10.17533/udea.iee.v42n1e02

**Published:** 2024-04-22

**Authors:** Carmen de la Cuesta Benjumea

**Affiliations:** 1 RN, MSc, PhD. Honorary Collaborating Professor, Department of Health Psychology. Universidad de Alicante. Alicante. Spain. Email: ccuesta@ua.es Universidad de Alicante Department of Health Psychology Universidad de Alicante Alicante Spain ccuesta@ua.es

**Keywords:** health research evaluation, research, nursing research, qualitative research, quality control., control de calidad, evaluación de la investigación en salud, investigación, investigación cualitativa, investigación en enfermería., avaliação da pesquisa em saúde, controle de qualidade, pesquisa qualitativa, pesquisa, pesquisa em enfermagem.

## Abstract

This article deals with the particularities of the quality of qualitative research, under the double lens of valuing it and ensuring it. While achieving the quality of qualitative research concerns only those who have opted for this methodology, assessing it is everyone's business because researchers in training will encounter, in the literature reviews, qualitative studies on which they must reflect and estimate their quality. Appreciating the quality of a research work is a complex activity as it is situated within a context and conducted by individuals who use any of the means available to do so. The means they use are criteria as evaluation guides and criteria checklists. For researchers in training, I suggest some guiding criteria to evaluate qualitative publications and ensure quality during the research process, key issues that they must address.

## Resumen

Este artículo trata sobre las particularidades de la calidad de la investigación cualitativa, bajo la doble lente de valorarla y asegurarla. Mientras que alcanzar la calidad de una investigación cualitativa atañe solo a los que han optado por esta metodología, valorarla es asunto de todos, ya que los investigadores en formación se encontrarán en las revisiones bibliográficas con estudios cualitativos sobre los cuales deberán reflexionar y estimar su calidad. Apreciar la calidad de un trabajo de investigación es una actividad compleja ya que está situada en un contexto y llevada a cabo por personas que usan alguno de los medios disponibles para hacerlo. Los medios que usan son los criterios como guías de evaluación y los listados de verificación de criterios. Para los investigadores en formación sugiero unos criterios guía para la valoración de publicaciones cualitativas y para asegurar la calidad durante el proceso de investigación, unas cuestiones claves a las que deben atender.

## Resumo

Este artigo trata das particularidades da qualidade da pesquisa qualitativa, sob a dupla lente de valorizá-la e garanti-la. Embora alcançar a qualidade da pesquisa qualitativa seja do interesse de quem optou por esta metodologia, Avaliar é tarefa de todos, pois os pesquisadores em formação se encontrarão em revisões bibliográficas com estudos qualitativos, sobre os quais deverão refletir e estimar sua qualidade. Avaliar a qualidade de um trabalho de investigação é uma atividade complexa, pois está situada num context e é realizada por pessoas que utilizam qualquer um dos meios disponíveis para o fazer. Os meios que utilizam são critérios como guias de avaliação e listas de verificação de critérios. Aos pesquisadores em formação, sugiro alguns critérios norteadores para avaliar publicações qualitativas e para garantir a qualidade durante o processo de pesquisa, questões chaves que devem ser abordadas.

## Introduction

Research is the backbone of a PhD program and, in many cases, master's programs; its quality is something that concerns us all, professors, directors of thesis or master's thesis and students, given that the advancement of knowledge and the success of researcher training depends on it. While achieving quality of qualitative research concerns only those who have opted for this methodology, evaluating it is a matter of all researchers in training, given that in literature reviews they will find qualitative studies about which they must reflect and estimate their quality. Today, it is expected that, theoretical frameworks or literature reviews and the justification of any study to include qualitative knowledge, given that if not done, the work will be incomplete. What is worse, in the case of quantitative studies, there will be no evidence that highlights the relevance of the research question, or which permits designing a measurement instrument according with the reality of the individuals; likewise, in intervention studies, qualitative knowledge provides essential information about the context in which said intervention will be implemented. Hence, this article deals with the particularities of the quality of qualitative research, under the double lens of evaluating and achieving it. 

To favor comprehending this work, the first thing I propose is that the appreciation of the quality of a study is subject to the paradigm on which said study is based. Thereafter, I explain that evaluating quality is a subjective activity situated within a context, given that it is carried out by people and not by instruments. In the appreciation of quality, I will focus on aspects researchers in training must look for and know how to appreciate. I will conclude by addressing those who are starting a qualitative study or are already undertaking one, and will propose the need to ensure quality during the research process itself. I have written about quality,^1^ now I center my attention on the key issues of its evaluation and achievement.

### The paradigm

I believe nobody is alien to the idea of paradigm, which is usually understood as a revolution in the way of thinking about something that leads to changes. In effect, one of the best definitions I know of paradigm is that which explains it as a set of beliefs that guide action.^2^ In research, these beliefs are based on a group of interconnected assumptions: the ontological, relating to what is believed about reality; the epistemological, about the relationship between the research and that which can be known; and the methodological, which refer to beliefs about how knowledge is obtained about the world. The paradigm defines for researchers that which they deal with, that is, legitimizes the research question and defines their task; that is, how they should act and the procedures they should use^2^. Hence, quality assessment is a paradigmatic issue and not a methodological or technical one. What is relevant is the perspective of the person evaluating; from this perspective, the evaluation criteria will emerge together with instruments that will be used and how these will be used. 

To prevent projects and qualitative studies from being judged with positivist criteria, in the 1980s Lincoln and Guba, in their text Naturalistic Inquiry,^3^ developed, among other seminal works, vocabulary and quality concepts of qualitative research. When I read this book for the first time, it seemed difficult to understand each concept; nevertheless, I was grateful that they had written it because it reaffirmed to me during my training as a researcher that what I was doing was scientific, although a different type of science. These authors explained that the validity of a qualitative study is achieved with confidence or trustworthiness and that for this the work, among other things, had to be credible, both in the methodological aspect and in its results. A language and concepts had been born to assess qualitative research. Years later, in an effort to strengthen scientific recognition, authors such as Tina Koch propose the equivalence of the concepts included in the criterion of trustworthiness with positivist criteria.^4^ For example, credibility was equated with internal validity and transferability was equated with external validity. This marked a milestone because it set the rigor of qualitative research on par with that of quantitative research, so that we were different among peers. 

Since then, much has been written and published about quality. This theme of constant interest in methodological development has not been free of debates and tensions.^1^^,^^5^ We could say that at the beginning attention centered on developing our own quality criteria and then taking care of promoting the quality of the research to be included in methodology manuals and, lastly, on developing means to assess it, coinciding with the movement of the evidence-based practice, with the expansion of publications of qualitative studies and with the growing need to conduct qualitative systematic reviews or meta-synthesis. 

The fact is that qualitative studies must be as rigorous as any research, and it must be taken into account that they have their own well-consolidated parameters. If researchers do not take this into account and expect, for example, for the results to be objective and extrapolatable, In addition to being unfair, their evaluation will possibly be wrong and taking as good what is not or ignoring what is valuable because it does not meet inappropriate standards. The evaluation is a challenging activity, especially because it is very easy to see defects in a work or to fall into purist and unempathetic positions that prevent recognizing the good and the meritorious.

It is true that, from that published about a topic, we can find marvelous studies that open doors for us to strengthen knowledge and others of little value. Thus, Sandelowski and Barroso^6^, in the systematic review on HIV and AIDS, found that qualitative publications could range from not being research due to not having results, to being confused with qualitative research due to presenting quantified and not described results. According to these authors, true qualitative studies, in turn, could have different conceptual levels, thus, from lowest to highest they found: the exploratory ones that were basically limited to stating the identified themes; the descriptive ones that developed them; and the explanatory ones that established new relationships among these themes. This range is determined by the conceptual proximity of the results with respect to the data, that is, the depth of the analysis. This already makes clear the need to assess the evidence, regardless of how challenging it may seem, especially when building the theoretical argument of our research. But what does this process entail? I will explain it ahead.

### The complexity of evaluating qualitative research

Evaluating a qualitative study is a complex activity because it implies diverse interconnected elements: the research report, the evaluation context, the person evaluating, and the means to do so. Each of these aspects will be explained. The document evaluated is a text created with a particular purpose and always with the aim of producing an impression. Sandelowski^7^ already indicated that research reports, whether theses or articles, are not minutes of what occurred, but the artifacts constructed. So, what we evaluate is a version and - generally incomplete - of what took place. Limits exist about what can be stated, written, and - of course - there are word limits. Due to such, when evaluating a work, if we notice any void, or a given canon is not complied, we should not assume it as a failure; first, we will think that it was not included in the report and we will decide the importance of this omission, we will also reflect on whether the canon not met has to do with other things, like the level of analysis presented or that quite simply the precept is for angels and not for human researchers. A reviewer of a manuscript I submitted some time ago for publication noted that the categories were not saturated as the manuals of the time indicated. The observation was correct to a certain point, given that the saturation of a category in practice is not an absolute term; the reviewer did not take this into account when strictly adhering to the definition of the concept to the letter. 

This anecdote brings us to the second issue, which states that the evaluation does not take place in a void, but within a context that will grant it sense. Thus, for the proposal of a research project, we will assess qualitative articles to develop an argument that will support the project, seeking sound and convincing evidence on the study topic. In the area of health, extensive documentation is available on the subjective experience of complex health-disease processes, on complications in the development and implementation of health services or interventions, and on expert knowledge in practice.^8^ We have high-quality qualitative theory that must be used; notable for its current relevance is the wealth of qualitative knowledge on chronicity and dependence pioneered by Charmaz.^9^^,^^10^

Currently, unlike other times, the amount of information available and accessible contrasts broadly with the difficulty present prior to being able to access such, particularly to qualitative studies that were not many and were disperse. I recall that during my PhD formation I travelled by train to another city to consult the collections of its university library and more than once returned empty-handed. Yes, I also wonder, how did we live without the internet? Most likely, in a few years we will ask ourselves how we survived without artificial intelligence!

Today, everything is connected and much is published, so search engines in databases can yield hundreds of references that we must screen for information to be manageable. In that respect, I only wish to state that establishing a date of publication as limit, such as the last five years, to retrieve works about our topic of interest, in the case of qualitative studies, should not be used exclusively, given that good qualitative evidence transcends time, that is, it does not expire. For example, if I am conducting a literature review for a study about palliative care and ignore the work of over 40 years by Quint Benoliel^11^about caring for a dying patient I am losing valuable information. Interpretive evidence accumulates in a connected and non-hierarchical way.

Upon retrieving information, we must discern that with the highest quality and relevance for the research we propose. In addition, given that in the area of health, disciplines, like nursing, are practiced, we must not lose sight of the practice context and must ask ourselves for the potential of the works we are evaluating to improve it. Herein, the assessment context will be academic and clinical. Besides being an activity situated within a context, the evaluation is subjective, eruditely subjective we could say. Those of us who evaluate have a certain training and methodological tastes that influence on the evaluation process,^12^ thereby, this requires that we keep in mind our preferences during the evaluation.^7^ Evaluating is, thus, a matter of passing judgment mediated by our subjective appreciation.^1^ At this point, it is clear that those of us who evaluate must, at least, be familiar with qualitative methodology besides being fair in our judgment: we should distinguish between significant errors and those that are not.^7^ Appreciation is, therein, based on experience and on methodological knowledge.

It is true that different evaluators can have different appreciations of the same work, and this has happened to many of us with the evaluation of manuscripts for publication. Aside from the confusion that this may cause, the issue in evaluation is not unanimous opinion, rather that assessments are informed and well-supported. Evidently, much of science is about persuasion, of convincing with logical and documented arguments.

Here, I must refer to the means to assess the quality of a qualitative report. Basically, two are used: criteria used as guide and criteria contained in checklists. Although general agreement exists on a study’s quality criteria, not all authors assign the same importance to each criterion, nor are all criteria included in the checklists. In addition, there are authors - who taking the pioneering work by Guba and Lincoln - introduce criteria of general application to any work, such as veracity or trustworthiness, transferability, congruence, and transparency,^13^ while others do so according to the research method - distinguishing, for example, the evaluation of a phenomenological study from an ethnographic one.^14^

Regarding the second evaluation means, there are closed checklists, and I wish to indicate that there are many, including the: Consolidated criteria for reporting qualitative research **(**COREQ),^15^ frequently used for publication in nursing journals, which has 32 items grouped into three domains: the research team, the study design, and the findings. Also, among those designed for the critical reading of qualitative studies within the evidence-based practice movement, there is the Critical Appraisal Skills Program Spain (CASPe) grid^16^ with 10 items and centered on the study results estimating their validity and applicability to the practice. These lists are useful for people with basic or introductory training in qualitative research, given that they contain quality criteria indicators and where they can be found in a text. However, because no consensus exists on the criteria that checklists should include, how to apply cut-off points and how to judge whether a study has met a standard, ^17^ the quality judgment is in the hands of the person evaluating the work and using a given list. Thus, the importance of the evaluator in determining the quality of a study is again highlighted. While using criteria requires evaluators to have greater experience and knowledge than checklists, they also require understanding and knowledge of qualitative research. 

In evaluating quality, as already seen, expert opinion comes into play and I base myself on it then, considering the training context of novice researchers, suggesting criteria as a guide. 

*Criteria to evaluate quality within the formative context*

The criteria I propose are some related to the product and others related to the research process (Table 1). My intention is not to add to what has been published, but to highlight that which I consider essential and, as a key, to keep novice evaluators from getting lost in the details and from being able to distinguish and appreciate what is relevant.

**Table 1:** Clues for quality of qualitative research

Assess Quality		Achieve Quality
Result criteria	Process criteria	Central issues
Evocative	Credibility	Interior point of view
Substantive relevance	Methodological/method coherence	Reflexivity
Credibility	Based on data	Time management

First, the criteria regarding the product. Considering the context of a research project in the area of health, a qualitative study must be *evocative* so that its results tie us with cases, experiences and situations of practice. Here, the evaluative vision is that of the clinicians, for whom the evocation resonates in their experience, in such a way that they achieve a more sophisticated or deeper understanding related to the practice. Hence, if upon consulting a work, this reaches us or impacts us, it is a sign of quality. The qualitative evidence of quality does not leave anyone who reads it indifferent, it moves and clarifies. For example, in the findings of a study of people with chronic kidney disease we concluded:^18^^)^

Chronic kidney disease and its treatment alters the feeling of who one is and what one who suffers from it can do. For people with chronic kidney disease *nothing is no longer like before* nor *are they who they were before*. The disease has disrupted their lives. However, they struggle to lead a life worth living in which the life provided by treatment is compatible with social, family, emotional and work life.

Besides being evocative, the study must contribute to what is known about the topic; due to such, we will value its *substantive relevance*, this means that we will examine the essential and the revealing that it contributes to what is already known about the topic. We will know how to recognize this because the work itself will indicate, in the discussion of the findings, what it adds to what is already known and, as informed readers on the subject, we will value it. If, on the contrary, the study indicates that it coincides with that presented in prior works, this is simply a verification or reiteration of what is known. 

A qualitative study must not only move, but also convince, so we will weigh its *credibility*, which is both for its findings and for the research process itself. Thus, we will estimate whether that proposed is plausible given the knowledge on the subject and if it is reasonable given the circumstances in which the study was conducted, such as its duration.^19^ In reality, qualitative studies take time, require prolonged periods of time in the field and unaltered analysis. Evaluation requires our distinguishing those reports that state that they did everything that had to be done, but without showing evidence of what they did.^7^ A case may be that it is reported that unstructured or semi-structured interviews were carried out and the interview guide presented contains many questions and/or that these are closed questions.

After this first filter focused on the research product, there are, in my opinion, the questions of the process, those that deal with evaluating the aspects related to how it was carried out. In this evaluation we must be cautious because, I know from experience, that many times aspects of the description of the method or methodology are sacrificed due to the word limits imposed by journals. Thus, some credibility aspect in the process may be threatened by these restrictions. For example, a study states that data collection was concurrent with the analysis and then does not show any indicator of this, such as that data collection was done in a staggered manner. In this case, as already indicated, the evaluator must weigh the importance of this omission within the work’s overall context. Regarding the process, besides credibility, the most important thing to assess is its *methodological coherence* or the method used. For example, that the objectives and the question coincide with the method chosen and this coincides with the data collection and analysis procedures. Likewise, we will verify that what is stated is *based on data*, that is, that the data analysis was inductive. To do this, the report must contain live data that clearly illustrate the concepts: when reading the live data, it immediately takes us to the concept. 

### Achieving the quality of the qualitative study

Although the qualitative methodology manuals and the criteria by which a work will be evaluated tell us in detail how we should do it, in this last part I would like to refer to three central issues that I must address as a researcher to achieve the quality of my study (Table 1). These are, to my understanding key, that will maintain the course of quality in the study, that will give meaning to what we do, avoiding ritualistic practices and, most importantly, will allow us to persevere the essence of qualitative research: that which appreciates the details and transmits universals. 

The first issue I propose is that of staying in the other's point of view, or *emic* point of view. Qualitative research is necessarily partial, It is concerned with showing things as they are from within, from the perspective of the person who lives or experiences them. Goffman, in the work on psychiatric patients, indicated that this is partiality essential to faithfully describe a situation, although adding that of being “exempted” from it as a matter of balance because almost everything written at the time about mental patients was done from the psychiatrist's point of view.^20^ With this explanation, Goffman aims at the heart of qualitative research, at what triggers it. Following this teaching, in a study I expose:

Thus, this study was motivated by gaps in the literature, the interest that as a nurse I have in family care, and the situation in Colombia where support for family caregivers, although necessary, is still scarce. Examining the strategies that caregivers develop in advanced stages of dementia, documenting the circumstances in which caregiving takes place and what effect this has on the course of the disease... reveals what we can and should do.^21^

Qualitative “bias” gives value to the experiences and points of view of those who live them and not of the experts who are outside such. Bearing in mind this research question and the study topic will help us remain in this vision throughout the study. A sign that we are entering the experience of the interior is when, for example, when transcribing an interview and it seems to us that it does not say anything relevant, it is likely that they do not say anything of what we expect to hear and therefore we do not recognize it. Here, it is fundamental to consider that qualitative research is about discovering and not about verifying what is already known. We will know that we have grasped the insider's point of view when the study participants tell us something like "I wouldn't have said it like that, but that's what happens" or they say "that's not my case, but it could be like that." Clearly, this is different from, for example, participants confirming that they have said what they have said in an interview, in this case we will not be entering the experience of the interior, but rather we will remain on its surface. It is the intensive and deep data analysis along with focused questions that will reveal the perspective of the interior, that is: the subjectivity of the experience. 

The second issue I propose is reflexivity. This consists of being aware of the effect that the research being conducted has on oneself, and the effect it produces on the study participants themselves.^1^ Here, I refer to reflexivity as researcher in training, something that has gone unnoticed in specialized bibliography. This reflexivity involves becoming aware of our expertise as beginning researchers, which will allow us, when required, to make the necessary adjustments. For example, during my PhD formation I made the mistake of negotiating through a third party the access to a health center to start my fieldwork. This caused misunderstandings about who I was and what I sought. Those who received me confused my identity, thought I was visiting and provided me with a large amount and variety of information, much of it irrelevant to my study. In the following health centers that I went to for fieldwork, I negotiated access personally and have done so ever since. There are no misunderstandings about my identity or what I intend as a researcher, this helps me obtain relevant data for the study. Adjustments to the research process can be made, even to the research question. If we notice that such is not significant, we can change it, as illustrated in the following quote:

The question that initially guided this study was "How are women and girls handling the AIDS epidemic in Mozambique?" However, as data collection advanced, it became evident that AIDS was nothing more than an oppressive aspect of the women's lives. At that point, we needed a broader question to capture the complexity of the women’s experience. Therefore, the research question evolved toward “How do women handle gender oppression in Mozambique?” ^22^

Persisting with the initial question would have led to less relevant results. Adjusting contributed to its quality. Changing or accommodating the research question based on the fieldwork does not go against quality but, rather indicates that we have situated ourselves on the interior point of view. Therefore, to achieve quality we must be attentive to what we do and how we do it, keeping in mind the purpose of our study and the spirit of qualitative research. 

Moreover, while conducting qualitative research, a frequent mistake is that of our preconceptions. During an interview, a student once asked a principal caretaker to tell her what she had done when she got up in the morning, to which the caretaker responded: “I wish I had gone to bed!”. The good thing about mistakes like this is that they suddenly place you in the other's reality, in their experience. And this is a grand opportunity for analysis. Thus, the so-called “errors”, during the course of the research are opportunities for discovery, for learning, and for improving the very research process and its procedures.^23^In fact, qualitative research has the particularity of self-correction, it develops flexibly, adjusting to contingencies or mending errors. For such, researchers in training need not only have a good methodology base, but also to recur to texts and people of reference that help them to detect and correct mistakes. Qualitative design is emergent; qualitative researchers do not act by design, but by acting as such, we design our studies, that is, we accommodate it to the contingencies and opportunities of the fieldwork, the data analysis shows us the path to follow. This way of developing design is a mark of quality.

The third and last issue to achieve the quality of the study is time management. The quality of our work may be affected by poor time management. If we have no time for reflection, to try again, to make changes and, due to lack of time, we perform a hasty analysis, a poorly prepared hurried thesis writing, this condemns the quality of the study. We must be able to reconcile research and training with our other lives, family life, social life and, in many cases, professional life. In our first studies, we underestimate the time it takes to do things, such as gaining access to the field, let alone the time it takes to think, *i*.*e*., analyze the data. I wish to underscore that qualitative research has different times from those of quantitative research. For example, I advise reserving half the total time available for the study for analysis and to be quite realistic with the amount of data to obtain, given that, if we obtain more data than we can analyze, it is a waste of time; valuable time that we then have to take from somewhere else, jeopardizing the quality of our study because analysis is usually the first thing sacrificed when we lack time. Developing the schedule for a qualitative study is an exercise in practical realism. Thereby, the best qualitative study is that which can be carried out without compromising its quality over well-planned and invested time, time that will allow us to discover and enjoy.

To conclude, evaluating a qualitative study concerns everyone; without qualitative knowledge, research projects are incomplete. Evaluation is a paradigmatic and not a methodological issue, it is an activity located within a context and carried out by informed individuals who use any of the means available to do so. Evaluating quality, therefore, consists in issuing qualified judgment and is not merely the result obtained through a measurement instrument. Similarly, achieving quality during the research process requires formative reflexivity, that in which one is aware of being in a learning process. Professors want researchers in training to be good evaluators and better builders of knowledge. I hope with these clues to make the work easier.
